# Supplementary material for: Isolation of Bioactive Metabolites from Soil Derived Fungus-Aspergillus fumigatus
Source: Microorganisms. 2023 Feb 26;11(3):590. doi: 10.3390/microorganisms11030590 (PMC10053833; doi:10.3390/microorganisms11030590)
Supplement: Supplementary file 1 [file microorganisms-11-00590-s001.zip › microorganisms-2130710-supplementary.pdf]

microorganisms

MDPI

Supplementary Information

Isolation of Bioactive Metabolites from Soil Derived Fungus-*Aspergillus fumigatus*

Harman Gill [1], Ellen M. E. Sykes [2], Ayush Kumar [2] and John L. Sorensen [1,*]

[1] Department of Chemistry, University of Manitoba, Winnipeg, MB R3T 2N2, Canada
[2] Department of Microbiology, University of Manitoba, Winnipeg, MB R3T 2N2 Canada
* Correspondence: john.sorensen@uumanitoba.ca

Table of Contents

1. NMR spectroscopic data
   1.1 Spectroscopic data of compound 1…………..2–4
   2.1 Spectroscopic data of compound 2……………..5
2. Table S1: Reference fungal isolates used in the phylogenetics analysis of present study and their GenBank accession numbers 5
3. Table S2: Primers used for amplification and sequencing of ITS region…5

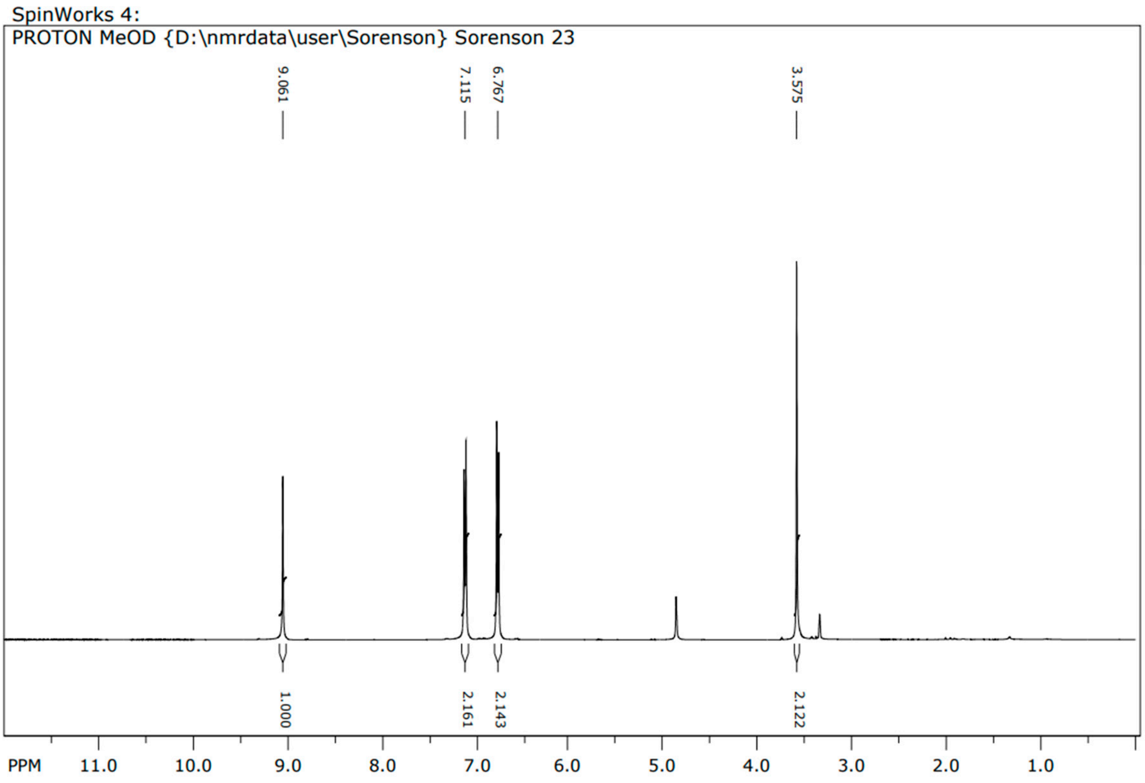

Figure S1. ^{1}HNMR spectrum of compound 1.

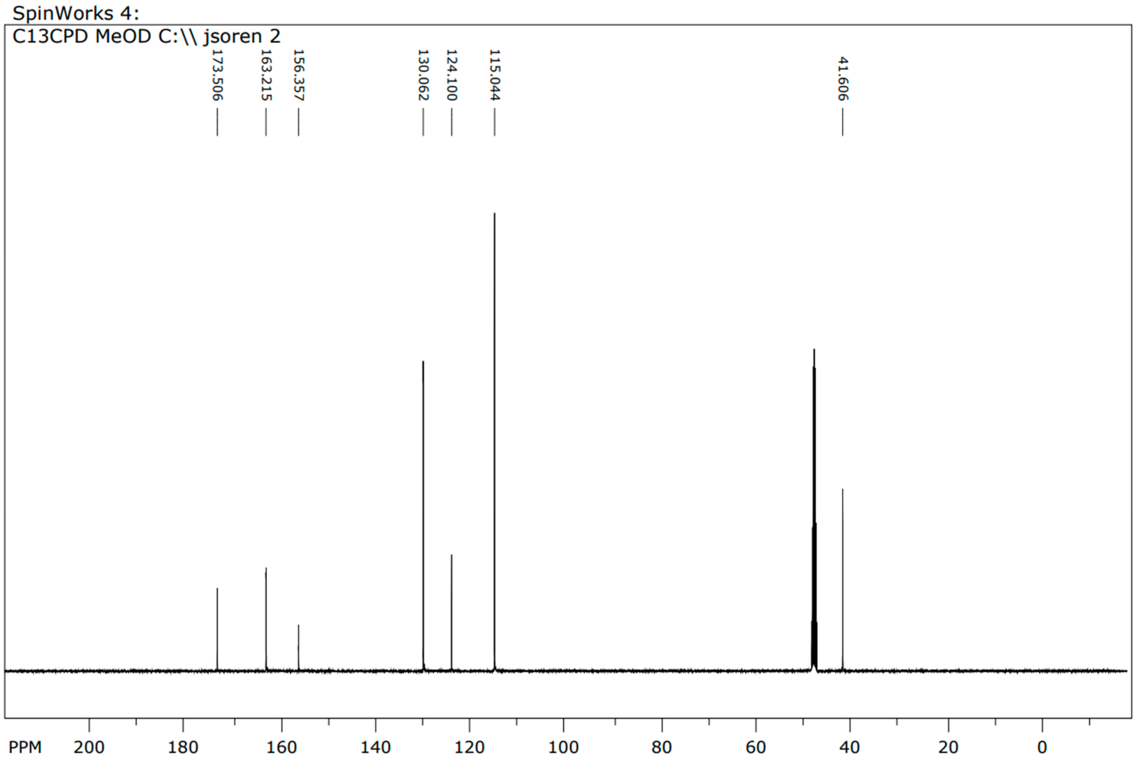

Figure S2. C NMR spectrum of compound 1.

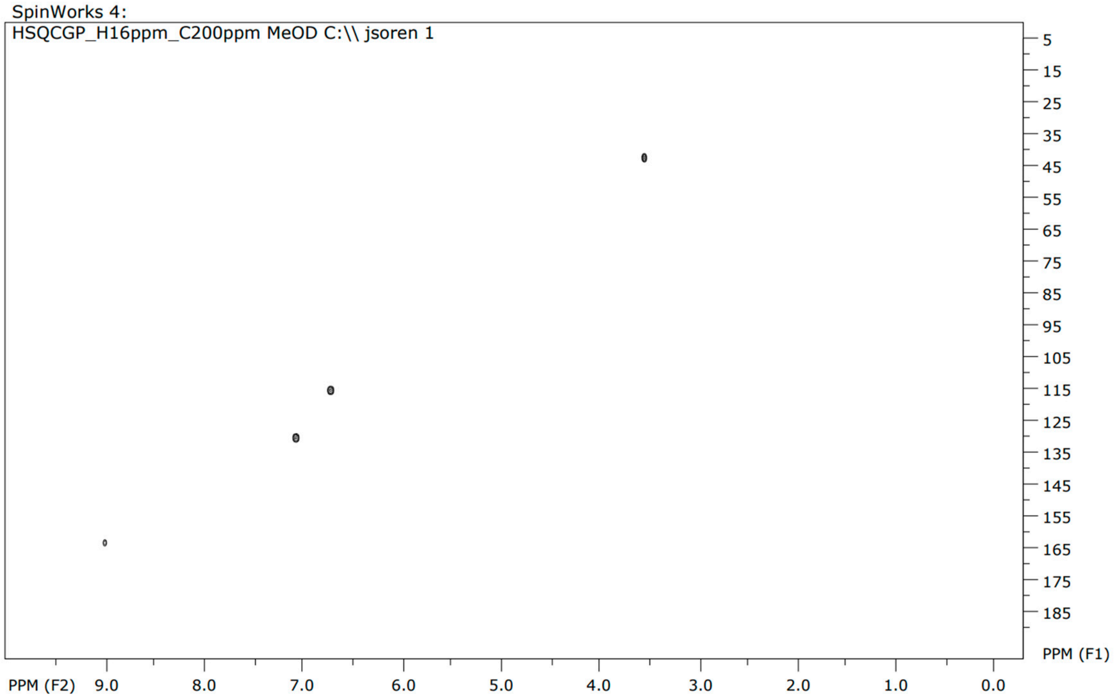

Figure S3. HSQC spectrum of compound 1.

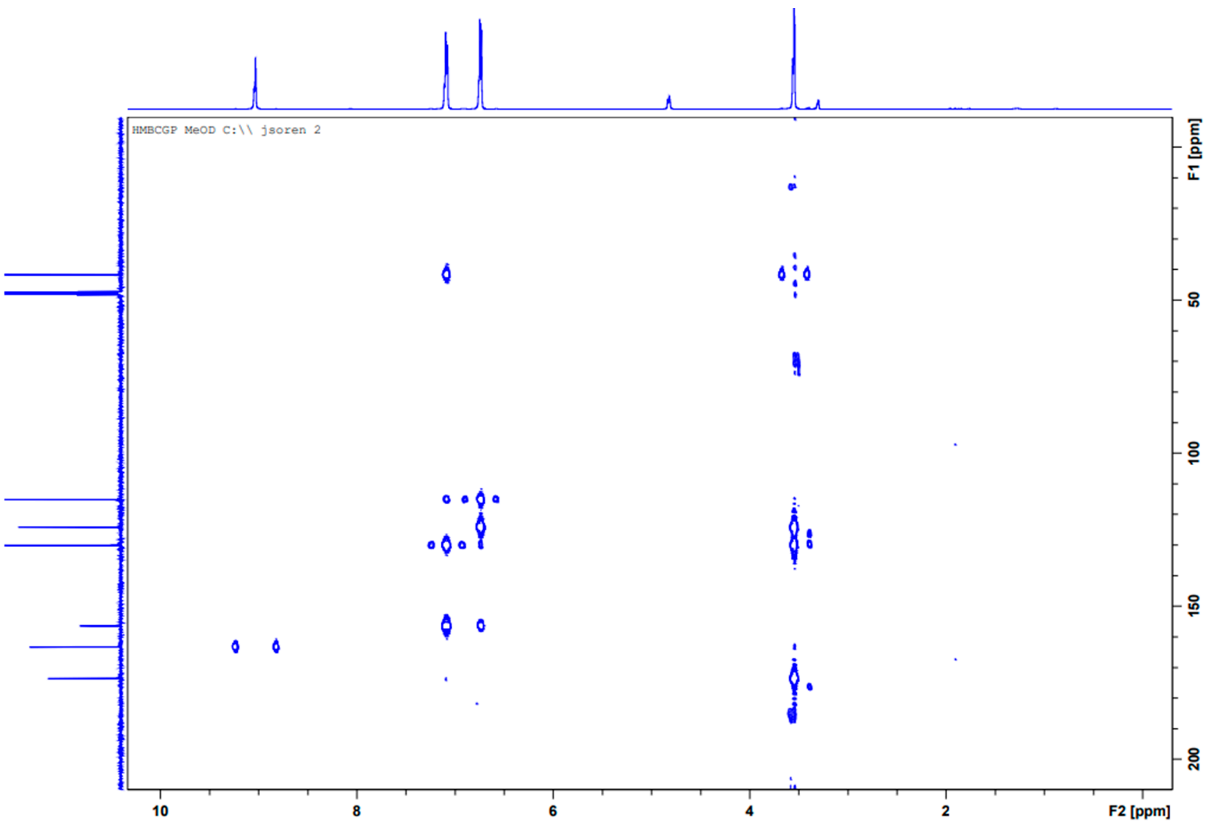

Figure S4. ^{1}H-^{13}C HMBC spectrum of compound 1.

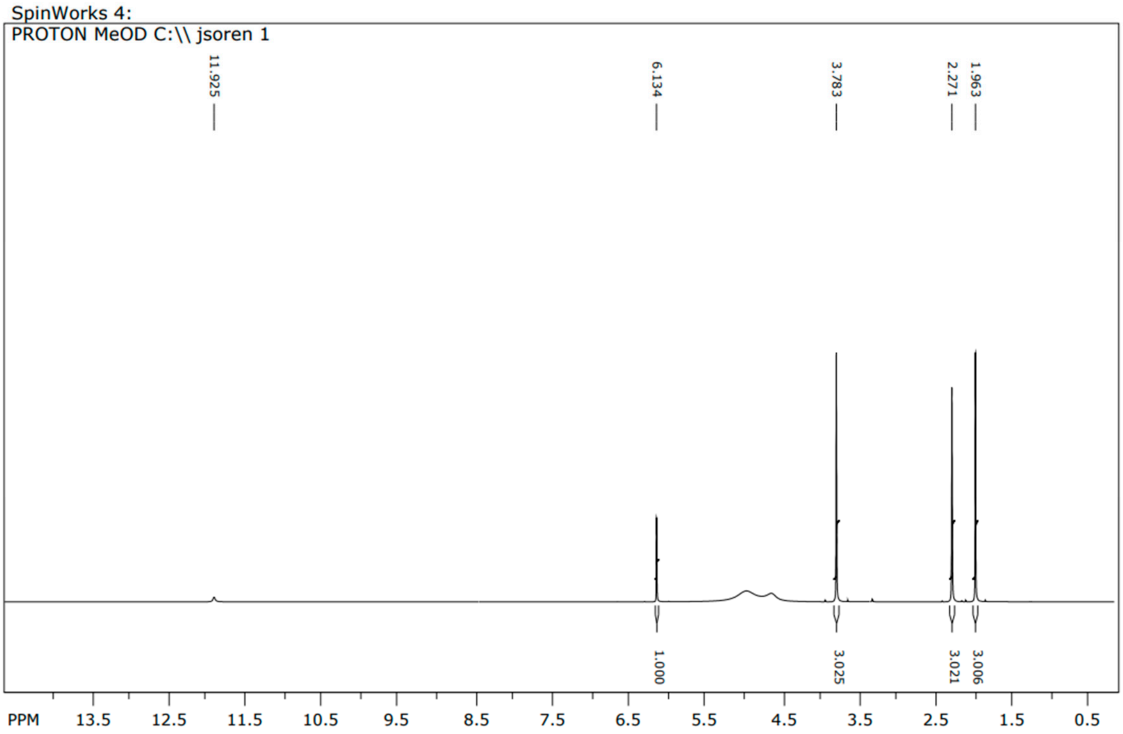

Figure S5. ^{1}H NMR spectrum of compound 2.

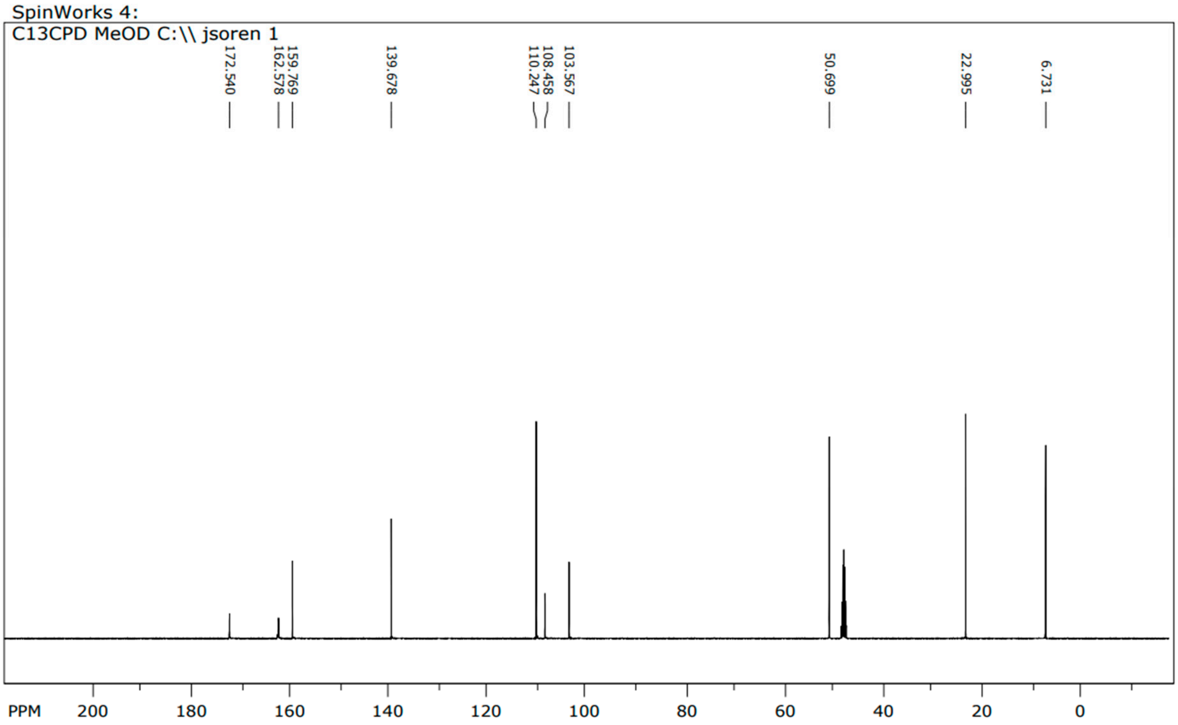

Figure S6. C NMR spectrum of compound 2.

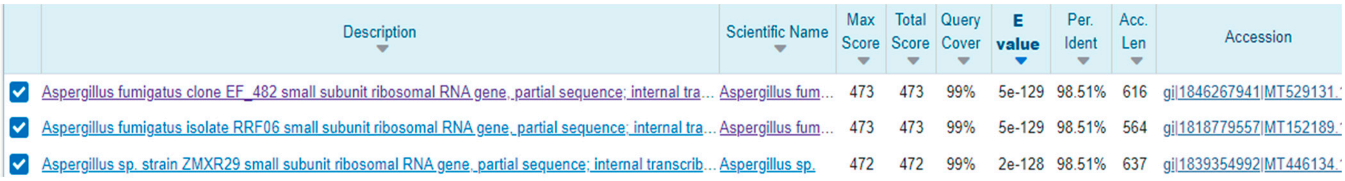

	Description	Scientific Name	Max Score	Total Score	Query Cover	E value	Per. Ident	Acc. Len	Accession
☑	Aspergillus fumigatus clone EF_482 small subunit ribosomal RNA gene, partial sequence; internal tra...	Aspergillus fum...	473	473	99%	5e-129	98.51%	616	gi|1846267941|MT529131.1
☑	Aspergillus fumigatus isolate RRF06 small subunit ribosomal RNA gene, partial sequence; internal tra...	Aspergillus fum...	473	473	99%	5e-129	98.51%	564	gi|1818779557|MT152189.1
☑	Aspergillus sp. strain ZMXR29 small subunit ribosomal RNA gene, partial sequence; internal transcrib...	Aspergillus sp.	472	472	99%	2e-128	98.51%	637	gi|1839354992|MT446134.1

Figure S7. Blastn search of S4, showed 99% query coverage and 98.51% with known *A. fumigatus* strain SL-2203.

Table S1. Reference fungal isolates used in the phylogenetics analysis of present study and their GenBank accession numbers.

S. No	Species	GenBank Accession
1	*Aspergillus fumigatus*	MT529131.1
2	*Aspergillus flavus*	NR_111041.1
3	*Aspergillus niger*	OM802854.1
4	*Aspergillus terreus*	MT436785.1
6	*Aspergillus ruber*	NR_131286.1
7	*Aspergillus fischeri*	NR_137479.1
8	*Penicillium sp.*.	KY930467.1

Table S2. Primers used for amplification and sequencing of ITS region.

S. No	Primer Code	Primer Sequence (5′ - 3′)
1	ITS1 F	TCC GTA GGT GAA CCT GCG G
2	ITS4 R	TCC TCC GCT TAT TGA TAT GC
